# PET tracers in glioblastoma: Toward neurotheranostics as an individualized medicine approach

**DOI:** 10.3389/fnume.2023.1103262

**Published:** 2023-02-27

**Authors:** Habibullah Dadgar, Narges Jokar, Reza Nemati, Mykol Larvie, Majid Assadi

**Affiliations:** ^1^ Cancer Research Center, RAZAVI Hospital, Imam Reza International University, Mashhad, Iran; ^2^ The Persian Gulf Nuclear Medicine Research Center, Department of Molecular Imaging and Theranostics, Bushehr Medical University Hospital, School of Medicine, Bushehr University of Medical Sciences, Bushehr, Iran; ^3^ Department of Neurology, Bushehr Medical University Hospital, School of Medicine, Bushehr University of Medical Sciences, Bushehr, Iran; ^4^ Department of Radiology, Cleveland Clinic, Cleveland, Ohio

**Keywords:** theranostics, neuro-Oncology, glioblastoma multiform (GBM), prostate-specific membrane antigen (PSMA), fibroblast activated protein (FAP), somatostatin receptors (SRS), chemokine receptor-4 (CXCR4)

## Abstract

Over the past decade, theragnostic radiopharmaceuticals have been used in nuclear medicine for both diagnosis and treatment of various tumors. In this review, we carried out a literature search to investigate and explain the role of radiotracers in the theragnostic approach to glioblastoma multiform (GBM). We primarily focused on basic and rather common positron emotion tomography (PET) radiotracers in these tumors. Subsequently, we introduced and evaluated the preclinical and clinical results of theranostic-based biomarkers including integrin receptor family, prostate-specific membrane antigen (PSMA), fibroblast activated protein (FAP), somatostatin receptors (SRS), and chemokine receptor-4 (CXCR4) for patients with GBM to confer the benefit of personalized therapy. Moreover, promising research opportunities that could have a profound impact on the treatment of GBM over the next decade are also highlighted. Preliminary results showed the potential feasibility of the theragnostic approach using theses biomarkers in GBM patients.

## Introduction

Glioblastoma Multiforme (GBM), which develops from glial cells, astrocytes or oligodendrocytes, is a heterogeneous invasive brain tumor that causes death in the ﬁrst two years after diagnosis. According to the World Health Organization (WHO), brain tumor classification has been updated to include genotypic markers, histological markers, and grade (1). A low survival rate and very few treatment options for GBM make it a particularly acute health challenge.

There are few effective treatments for primary brain tumors despite significant advances in revealing their molecular underpinnings over the past decade. The most important barriers to developing effective treatments are the tumor blood-brain barrier (BBB), intra-, and intertumoral heterogeneity, and intrinsic resistance to chemotherapy and radiotherapy (2, 3). A number of strategies are currently being investigated including immunotherapy, gene therapy, and novel drug-delivery technologies that bypass the blood-brain barrier. A combination of therapies may also be necessary to achieve a broad, durable antitumor response.

Targeted therapies play an important role in modern treatment concepts. Additionally, the molecular heterogeneity of the tumors, including GBM, also makes this entity an ideal candidate for individualized and targeted treatments (4). To enhance patient selection and predict patient responses to treatment, nuclear imaging appears to be an important component of patient care and personalized medicine. Through a theragnostic approach, as an innovative approach in this field, radiotracer pairs with identical chemical and biological characteristics (but labeled with different isotopes) are used for both diagnosis and therapeutic purposes. In order to develop a “theragnostic” agent, it is necessary to combine targeted therapy with diagnostic imaging, such as scintigraphy, single photon emission tomography (SPECT), SPECT/computed tomography (CT), positron emotion tomography (PET)/CT, and/or PET/magnetic resonance imaging (MRI), in order to identify patients who may benefit from the treatment. Imaging systems that use specific theranostic probes allow physicians to visualize and assess disease targets and control or eliminate them as appropriate (5). To date, many PET agents have been developed for GBM. The already-established PET tracers are focused on general cancer hallmarks that are not specific to any tumor type. Most of them are sustained proliferation markers that indicate an increase in glucose metabolism, protein synthesis, or DNA replication. Neurotheranostics aims to revolutionize staging and improve diagnostic and therapeutic outcomes for neurological disorders. This review comprehensively addresses PET radiotracers and recent advances in neurotheranostics for GBM including integrin receptor family, prostate-specific membrane antigen (PSMA), fibroblast activated protein (FAP), somatostatin receptors (SRS), and chemokine receptor-4 (CXCR4). Moreover, promising research opportunities that could have a profound impact on the treatment of GBM over the next decade are also highlighted.

## Glucose metabolism PET

While differentiation between primary brain tumor from metastasis is problematic using conventional imaging, ^18^F-fluorodeoxyglucose (^18^F-FDG) PET may be helpful in depicting involved lesions or localizing the primary cancer site (6, 7). Heterogeneous primary brain tumors may show low or high uptake, especially in GBM with necrosis. For instance, astrocytomas and gangliogliomas show relatively high FDG uptake despite their low grade (8). To overcome the greatest clinical challenge of ^18^F-FDG PET in brain tumor imaging, delayed ^18^F-FDG imaging can sometimes improve discrimination between tumor and normal lesion due to prolonged radiotracer retention in the tumor relative to the gray matter and radio-necrosis (9, 10). It has been revealed that in low-grade tumors, the FDG uptake is similar to the white matter, while the uptake can be in the range of the normal gray matter in high-grade tumors. Generally, the tumor to white matter uptake ratios greater than 1.5 or the tumor to gray matter uptake ratios greater than 0.6 are used to differentiate benign tumors (grades I and II) from malignant tumors (grades III and IV) (11). Likewise, contrast-enhanced (CE)-MRI using gadolinium cannot reliably distinguish active tumors from radio-necrosis (12). Therefore, PET/MRI may indicate the active metabolic margins of the tumor as a guided biopsy procedure as well as correct tumor delineation during treatment planning and checking treatment response (13, 14). FDG PET/MR imaging with and without CE has higher specificity (97%) compare to MRI scan alone (23%) for the detection of recurrence, and higher FDG uptake in the tumor with recurrent glioma indicates a worse survival (15). Medulloblastomas as malignant brain tumors have high FDG uptake (16, 17).

According to the RANO guidelines, amino acid PET is superior to FDG PET imaging; therefore, the use of amino acids PET is recommended against ^18^F-FDG for brain tumor imaging (18).

## Amino acid PET tracers

Due to the high tumor-to-brain contrast in malignant tissues and the low uptake in normal brain tissue, radiolabeled amino acid tracers are in the first line of brain tumor imaging, including O-(2-^18^F-fluoroethyl)-L-tyrosine (FET) (^18^F-FET), Carbon-11-methyl-L-methionine (MET) (^11^C-MET), 3,4-dihydroxy-6-^18^F-fluoro-L-phenylalanine (FDOPA) (^18^F-FDOPA), *α*-[11C]Methyl-l-tryptophan (AMT) (^11^C-AMT), and ^18^F-fluciclovine (^18^F-FACBC) (19–24). Tumor delineation is highly effective for planning stereotactic biopsies, resection, and treatment planning in radiotherapy (25–28). In this regard, ^11^C-MET and ^18^F-FET amino acid-based imaging modalities have a high accuracy for delineation of the tumor extent in non-enhancing gliomas (27). A disadvantage of these tracers is the small amino acid uptake (30%) in low-grade II gliomas; therefore, they do not exclude gliomas (29–31). Because of the short half-life of ^11^C (20 min) from ^11^C-MET in the clinical studies, ^18^F-FET was developed (32, 33) and used as its alternative with more sensitivity. ^18^F-FET PET combined with MRI may improve tumor diagnosis and increase speciﬁcity (21). Moreover, amino acid PET tracers provide valuable information for diagnosis and differentiation of radiation injury from treatment-related changes due to the tumor relapse (21, 34–37).

**Figure 1:**
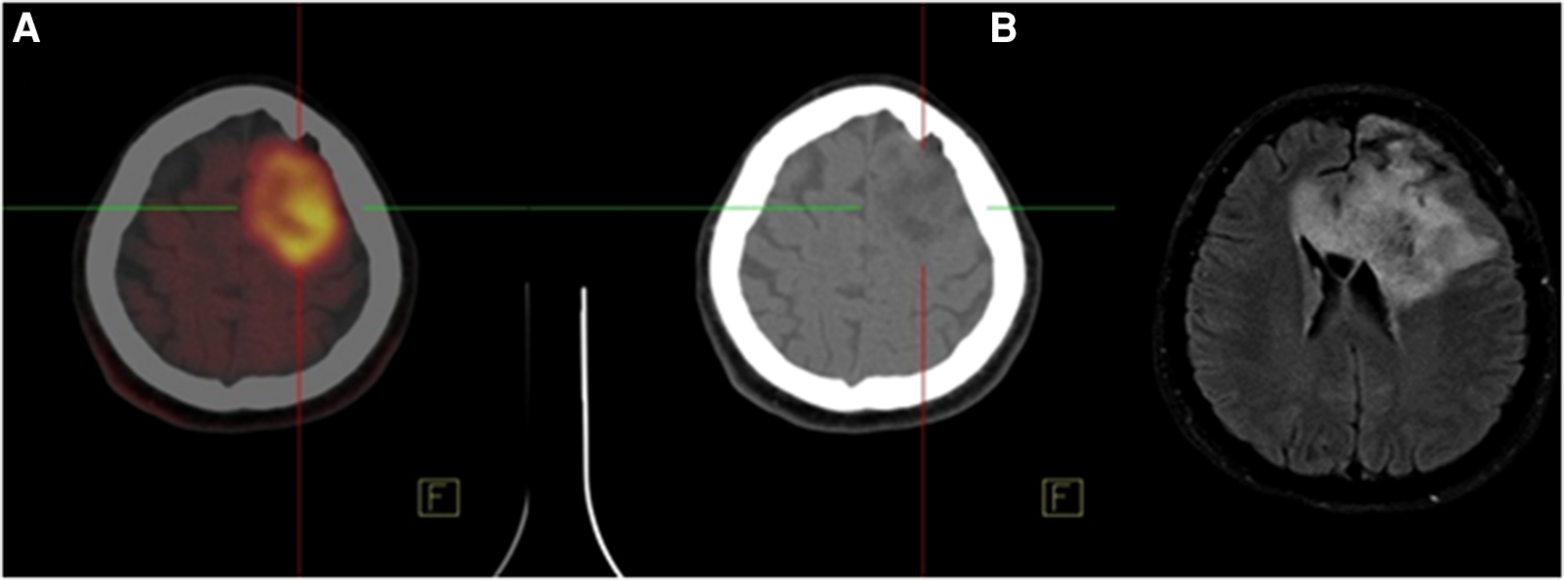
A 58-year-old man with high-grade glioma underwent brain ^18^F-FET PET/CT indicated intense radiotracer uptake (SUVmax = 7.68) in the left parietal lobe. (**A**) Accordingly, the tumor showed no change in Gd enhancement on FLAIR axial MRI with Gd. (**B**) Therefore, recurrence or residual disease was noted in the left parietal lobe.

FET-PET is superior to FDG-PET with higher sensitivity. The sensitivity is about 94% for high-grade tumors (WHO III-IV) and 68% for low-grade tumors (WHO I–II) for differentiation of high from low-grade gliomas by low uptake in the low-grade tumors (29–32, 38, 39). The diagnostic accuracy of ^11^C-MET is slightly lower than FET (11, 40), which is related to the higher affinity of ^11^C-MET for inﬂammation (37). FDOPA as a dopamine analog metabolize through monoamine oxidases or catechol-O-methyltransferase, it labels to ^18^F (^18^F-FDOPA) and used for neuroendocrine tumors (41) and neurological applications. A study found that FDOPA could diagnose the recurrence lesions with a sensitivity of 100% and specificity of 85.7% vs. 47.6% and 100% of FDG PET, respectively (42). Therefore, FDOPA is superior to ^18^F-FDG PET for assessment of recurrence in low-grade tumors while it makes no significant difference in high-grade gliomas (42, 43). A comparative study found no significant difference between FDOPA and FET in high-grade gliomas, while uptake ratios were 10%–15% higher for FET compared to FDOPA (44). High levels of ^11^C-AMT accumulate in gliomas *via* the Kynurenine pathway, which is responsible for producing NAD + from the degradation of tryptophan, an essential amino acid (45). In patients with positive recurrent glioma on MRI, AMT uptake by the tumor may differentiate recurrent glioma from radiation injury in PET study (46). Low FLT uptake in the brain cortex caused to diagnose the small malignant lesions (47). Previous investigations revealed that ^18^F-FACBC PET had a potential for use in primary staging in gliomas (48–52). In a preliminary study of 6 patients, Tsuyuguchi et al. found that ^18^F-FACBC might provide a better response than ^11^C-MET for the initial detection as well as the detection of high-grade recurrent gliomas (49). Unsurprisingly, the results of ^18^F-FACBC PET/MR imaging were significantly better than the results of ^11^C-MET PET/MRI (82% vs. 10%). In summary, compared to ^11^C-MET, ^18^F-FACBC has a higher detection rate in recurrent and progressing gliomas and their images have a better contrast due to the lower background in the normal brain cortex (51). Figure 1 illustrates a 58-year-old man with high-grade glioma who underwent brain ^18^F-FET PET/CT imaging. On ^18^F-FET PET/CT, there was intense radiotracer uptake in the left parietal lobe whereas T2-W MRI showed a lesion with no change in enhancement in post-contrast imaging.

In a systematic review of the recent studies, both amino acid PET tracers and perfusion-weighted imaging modalities were able to detect high-grade tumors, identify tumor recurrence, differentiate recurrence from treatment effects, and predict survival. Amino acid PET tracers performed better on these measures than perfusion-weighted imaging; however, they had the strongest results when combined. Studies of amino acid PET tracers with magnetic resonance spectroscopy (MRS) demonstrated that both modalities had diagnostic potential but MET PET and FDOPA PET performed better than MRS (53).

## New imaging targets

### Tumor hypoxia

Hypoxia is a main factor in treatment outcome and prognosis in different cancers, including glioma (54). Multiple radiotracers can be used to visualize hypoxia or sense oxygen levels. The tracer ^18^F-Fluoromisoinodazole (^18^F-FMISO) was developed for glioma imaging; however, its success is limited due to low sensitivity for differentiating normoxic from hypoxic tissue as well as a low blood brain barrier permeability (55, 56). Nevertheless, some results such as immunohistochemistry (IHC) findings, suggest that additional clinical studies are needed to evaluate ^18^F-FMISO as an imaging tool for hypoxia (57, 58). Moreover, the use of multiple PET radiopharmaceuticals is suggested to improve the planning for patient treatment and monitoring due to enhancing tumor detection and metabolic and genetic characterization. However, further research is required to eliminate the technical and logical issues of this technique and develop the concept of simultaneous multiplexing of ^18^F-FMISO with other PET oncology tracers (54). ^18^F-labeled flouroazomycin arabinoside (^18^F-FAZA) has the potential to be a promising alternative to ^18^F-FMISO offering an improved tumor-to-background ratio regarding faster blood clearance (59, 60). There is preliminary evidence that ^18^F-FAZA PET may be useful for assessing radiotherapy response in glioblastoma patients (61).

### Translocator protein (TSPO)

Like peripheral cancers, brain cancer cells trigger inflammation as part of the immune response. A wide range of neuroinflammatory conditions, including high-grade GBM, have been studied with translocator protein 18 kDa (TSPO) PET imaging (62–66). The TSPO, previously known as the peripheral benzodiazepine receptor, is highly expressed in GBM and plays a critical role in essential mitochondria-based physiological processes (67, 68). According to several studies, there is a positive correlation between TSPO expression and the grade of malignancy and glioma cell proliferation; however, it has a negative correlation with survival in patients with glioma (69–71).

### Cellular proliferation

3′-deoxy-3′-fluorothymidine (FLT) was used as an antiviral compound in patients with HIV for the first time (72). It's absorbed by cells and is subsequently phosphorylated by thymidine kinase 1 (TK1), which causes intracellular trapping within the cell. In this way, FLT retention within the cell provides a measure of TK activity, a key enzyme for cell proliferation (73). The increase in TK1 activity in normal cells occurs only during the DNA synthetic phase (72), while it occurs more and permanently in malignant cells. ^18^F-FLT uptake has a strong correlation between SUV of ^18^F-FLT and the proliferative status in lung cancer, colorectal carcinoma, non-Hodgkin's lymphoma, and melanoma (74–77). ^18^F-FLT is also considered to be an attractive PET tracer for the imaging of brain tumors due to the low uptake of ^18^F-FLT in the intact brain tissue and providing a low-background cerebral image (78). Furthermore, unlike ^18^F-FET, ^18^F-FLT requires a breakdown of the blood-brain barrier for it to be absorbed (79). Based on the results of a preliminary study in a low number patients with newly diagnosed gliomas, FLT uptake heterogeneity using textural features seemed to be useful for the assessment of proliferation and potential prediction of survival in these patients (80).

^18^F-FLT PET as a marker of cellular proliferation was compared with ^18^F-FDG in 25 patients with newly diagnosed or previously treated glioma. The results showed that ^18^F-FLT was more sensitive than ^18^F-FDG, correlated better with Ki-67 values, and appeared to be a promising tracer as a substitute marker of proliferation in these patients (81). For recurrent malignant glioma patients treated with bevacizumab and irinotecan, kinetic parameters from FLT provided sufficient information to predict the overall survival compared to those from FDOPA (82). A meta-analysis study indicated that ^18^F-FLT PET had a moderately better overall accuracy for diagnosing glioma recurrence compared with ^18^F-FDG (83).

Fernandez et al. determined the probability of combining ^18^F-FLT PET and MRI to improve the detection of tumoral tissue compared to MRI alone in 13 patients with newly diagnosed glioma. They also evaluated whether ^18^F-FLT uptake had a prognostic value by studying its association with histopathological features. According to the results, a combination of MRI and ^18^F-FLT PET detected additional tumor tissue, which might lead to a more complete surgical resection. The negative predictive value of a negative PET was also increased when combined with a negative MRI. Nevertheless, ^18^F-FLT underestimated the margins of the lesion and did not correlate with histopathological findings (84).

According to Nikaki et al. ^18^F-FLT accumulates in metastatic lesions, and it is highly sensitive and accurate. They reported that it could be used as a complementary tool to other imaging procedures. The tumor-to-background ratio and SUVmax provided the best diagnostic accuracy, while PV50% (proliferation × volume) and PV75% were of less diagnostic significance (85).

### Poly ADP-ribose polymerase (PARP-1)

One of the most abundant isoforms of PARP enzymes is nuclear enzyme poly(ADP-ribose) polymerase 1 (PARP-1) (86). Among the 18 members of the PARP family, PARP-1 is the founder and major isoform with diverse regional expression, subcellular distribution, and domain composition with mono (ADP-ribosylation) and poly (ADP-ribosylation) activities (87).

Several physiological functions of Poly(ADP-ribosyl)ation (PARylation) have been implicated, including DNA repair, gene transcription, cell cycle progression, cell death, chromatin function, and genomic stability (88). Since PARP becomes activated in response to DNA breaks, understanding how various endogenous species can induce DNA strand breaks, and thus activate PARP, in various diseases has become increasingly important. Several studies have demonstrated that neutralizing peroxynitrite and/or inhibiting PARP pharmacologically or genetically is effective in treating cardiovascular, inflammatory, vascular, and neurodegenerative diseases by preventing cell death as well as down-regulating multiple inflammatory pathways (89–91). Despite PARP1's therapeutic potential, it has also attracted attention as a target for imaging agents. Reiner et al. developed a PARP agent for PET/CT imaging based on the Olaparib scaffold using rapid bioorthogonal conjugation chemistries (^18^F-BO) (92). ^18^F-BO (biorthogonal olaparib) was produced as the ﬁrst inhibitor for PET imaging. Another specific target, ^18^F-PARPi, has been successfully used in GBM cell lines with high binding to peripheral tumors. It has been shown that the biodistribution and uptake of this tracer in the brain is very low around 2 h post-injection. In comparison to previous amino acid-based tracers, PARPi has a lower uptake in brain tumors with a lower background uptake in the cortex (93). As an important point, small PARPi molecules are needed for penetrating through the BBB as a major obstacle for any successful radiotracer targeting GBM. This limitation is mostly related to poor BBB penetration and high chemoresistance of GBM cells (94). With a high precision and good signal/noise ratio, ^18^F-PARPi can be used to visualize glioblastoma in xenograft and orthotopic mouse models. It offers new opportunities to image tumor growth and monitor interventions non-invasively (95).

^18^F-PARPi-FL, as a bimodal fluorescent/PET agent, is used for PARP1 imaging in glioblastoma cells. A study found that PARPi-FL could be radiolabeled and used to augment the limitations of the “fluorescence only” intraoperative imaging molecule (96). For this purpose, PET and SPECT imaging were performed in orthotopic glioblastoma models with ^124^I- and ^131^I-I2-PARP inhibitor (Olaparib) (97). The results showed the specific binding of the I2-PARPi tracer to PARP1 indicating the potential of this tracer for glioblastoma detection. Moreover, the results of an important preclinical study by Pirovano et al. provided the first full characterization of an auger-emitting PARP inhibitor, which showed a survival benefit in mouse models of GBM and verified the high potential of ^123^I-MAPi for clinical translation (98). With their short range of action, auger electrons possess high biological efficacy, which allows them to inflict DNA damage and cell death precisely. In addition to imaging tumor progression and monitoring therapy response, ^123^I-MAPi can also be used to calculate patient dosimetry.

### Sigma-1, sigma-2

Human tumor cell lines including C6 glioma, neuroblastoma, and NG108-15 neuroblastoma-glioma hybrid express sigma 1 and sigma 2 receptors. Sigma 1 receptors are associated with invasiveness, while sigma 2 receptors are associated with proliferation. These two receptors are interesting targets for PET imaging in glioblastomas (99). Initially, ^11^C-SA4503 sigma-1 based tracer was prepared as the ﬁrst PET ligand for GBM (100). Moreover, ﬂuorinated sigma-1 receptor ligands were developed such as ^18^F-(S)-Fluspidine, ^18^F-FTC-146 and ^18^F-FPS with high binding affinity. Further efforts by radiopharmacists led to ^125^I- PIMBA and ^18^F-RHM-4 with high background binding to S2R (101, 102).

### Epidermal growth factor receptor

Several malignancies have been associated with the human epidermal growth factor receptor (EGFR) family. All four members of this family, including HER1 (EGFR, ErbB1), HER2 (Neu, ErbB2), HER3 (ErbB3), and HER4 (ErbB4), play an important role in cell growth regulation, proliferation, and tumor migration (103). Epidermal growth factor receptor overexpression and activation of downstream signaling pathways, including RASYRAFYMAPK and PI3K/AKT, is considered to play a critical role in developing and progressing malignant gliomas (104, 105). Therefore, it is imperative to develop various treatment options, including tyrosine kinase inhibitors and monoclonal antibodies targeting EGFR, HER2, or both. Additionally, molecular imaging radiopharmaceuticals targeting EGFR family can improve the sensitivity and specificity of glioma detection (106). Following the use of ^64^Cu-DOTA-trastuzumab PET in breast cancer patients, it was found to be effective in detecting brain metastases (107). As part of a pilot study conducted in 2014, 11 patients with histopathologically proven GBM underwent PET/CT examination before surgery using ^11^C-PD153035. Six of the 8 patients with GBM were clearly visualized by ^11^C-PD153035 PET/CT, while 2 patients with GBM, 1 with anaplastic astrocytoma, and 2 with oligodendroglioma did not show any significant uptake. According to the results, ^11^C-PD153035 PET/CT was a promising tool for EGFR-targeted molecular imaging of GBM, which could translate into clinical applications to select patients suitable for EGFR-targeted therapies and to assess the early response of malignant gliomas to such therapies (108). A recent study evaluated the potential use of ^89^Zr-DFO-nimotuzumab in assessing the EGFR status in glioma (109).

## Potential theranostics-based receptors

### Integrin receptor family

Integrins are heterodimeric transmembrane glycoproteins containing 24 *αβ* (18 different *α* and 8 *β*) subunits that regulate cell survival, proliferation, and differentiation through their ability to transduce signals and interact with other cellular receptors [Table 1] (110).

**Table 1:** Summary of integrins involved in GB.

Integrin Type	Roles in GB	Reference
*α*vβ3	Migration Invasion Angiogenesis Survival Therapy resistance Prognostic marker	(111–114)
αvβ4	Proliferation Therapy resistance	(115, 116)
αvβ5	Migration, Invasion Angiogenesis Therapy resistance	(117, 118)
αvβ8	Invasion Angiogenesis	(119, 120)
α3β1	Migration, Invasion Stemness Prognostic marker	(121–123)
α5β1	Proliferation Migration Invasion Survival Therapy resistance	(113, 114, 121, 124)
α6β1	Invasion Stemness	(111, 117, 125)
α9β1	Migration Angiogenesis	(122, 126)

Among them, the pro-angiogenic *α*v*β*3 has been found to be highly expressed in high-grade brain tumors (112). This integrin belongs to the subclass of integrins that recognize the tripeptide sequence Arg-Gly-Asp (RGD) and is found in numerous extracellular matrix proteins, including fibronectin. While *α*v*β*3 integrins were first introduced as a marker of tumor progression and angiogenesis, specifically on both tumor-associated EC and GB cells, *α*v*β*5 is also used as a complementary attractive therapeutic target in GB (113, 114, 119, 127–129). Numerous studies confirmed the pro-tumorigenic and pro-angiogenic activity of *α*v*β*3/*α*v*β*5 and most recently *α*6*β*1 in GBM (130, 131), which makes them suitable for anticancer therapy (132). In fact, comparative IHC of integrins in GB has shown an overexpression of *α*2, *α*3, *α*4, *α*5, *α*6 and *β*1 (133) as well as *β*3/*α*v*β*3, *α*v*β*5, and *α*v*β*8 (134). Moreover, *α*v*β*3 is preferentially expressed at the invasion front of GBM. Integrins, as the main link between a cell and extracellular matrix, play an essential role in tumor invasion in the complex regulatory network of tumor progression (135). Due to its inherent safety and ability to target both tumor and endothelial cells, RGD peptide can be used as a selective carrier for delivering anti-cancer drugs. In this regard, a number of RGD peptide drug conjugates have been proposed as targeted drug delivery systems (136, 137). Radiolabeled cyclic RGD peptides in combination with radio-immunotherapy enhance therapeutic regimens with rapid and accurate detection of tumor growth and metastasis (138, 139).

Over the past decades, several radiotracers targeting *α*v*β*3 have been developed and investigated for clinical applications. Many of them are based on tripeptide RGD due to its high affinity and specificity for integrin *α*v*β*3. All of the investigated RGD peptides have shown very similar *in vivo* distribution with high uptake in the urinary tract, due to the urinary elimination of the tracer, as well as moderate liver and intestine uptake. In one study, a sugar amino acid was conjugated to the cyclic peptide c(RGDfK) to design ^18^F-galacto-RGD PET tracer (140). In ^18^F-Galacto-RGD PET images, the tracer uptake significantly correlated with the amount of integrin 3 expressed in tumor samples based on IHC examination. The data suggested that ^18^F-Galacto-RGD PET might be a promising tool for planning and monitoring individualized cancer therapies targeting this integrin. A first-in-human study of ^64^Cu-labeled long-acting integrin *α*v*β*3 targeting molecule, ^64^Cu-NOTA-EB-RGD, indicated the safety, favorable dosimetry profile, and pharmacokinetic properties. In three GBM patients, the EB-derived albumin-binding moiety present in ^64^Cu-NOTA-EB-RGD resulted in more efficient pharmacokinetics and a higher contrast. Theranostic applications for ^64^Cu-labeled EB-RGD such as ^67^Cu, ^177^Lu, ^90^Y or ^225^Ac were recommended (141). Recently, the *in vitro* and *in vivo* characteristics of a ^68^Ga-radiolabeled dimeric RGD, ^68^Ga-NODAGA-c(RGDyK)2, were investigated in a glioblastoma multiforme tumor model. ^68^Ga-RGD peptides showed better imaging properties than ^18^F-FDG and ^18^F-FLT in a glioblastoma tumor model in mice. Except in the excretory organs, ^68^Ga-RGD peptides showed higher tumor-to-background ratios (142). A study evaluated the efficacy of ^68^Ga-NOTA-RGD dimer for noninvasive integrin *α*v*β*3 imaging in preoperative patients with different grades of glioma. According to the results, it could evaluate glioma demarcation more precisely compared to ^18^F-FDG PET/CT because of being specific to brain tumors, but not to brain parenchyma, except for the choroid plexus. Moreover, ^68^Ga-RGD SUVmax had a significant correlation with glioma grade (143). Lo et al. developed a long-circulation integrin-targeted molecule consisting of a DOTA chelator, a truncated Evans blue dye (EB), a modified linker, and cRGDfK peptide. ^111^In-DOTA-EB-cRGDfK showed significant tumor accumulation with a high tumor-to-background ratio at 24 h post-injection in the preclinical phase. The data suggested that the ability to specifically bind to *α*_v_*β*_3_-expressing lesions present in the radiolabeled DOTA-EB-cRGDfK could be considered as a promising factor for glioblastoma tumor imaging indicating its potential for use as a theranostic radiopharmaceutical (144).

### Chemokine receptor-4 (CXCR4)

Chemokine receptor-4 (CXCR4) was introduced as a member of the G-protein-coupled chemokine receptor family. It affects embryogenesis, neo-angiogenesis, hematopoiesis, and inflammation. In addition to hematological malignancies, solid neoplasias express CXCR4 chemokine receptors. The activation of CXCR4 by CXCL12 induces migration and/or survival of neoplastic cells, such as tumor cells from brain tumors (neuronal and glial tumors), colorectal cancers, prostate cancer, melanoma, renal cell cancer, neuroblastoma cells, and ovarian cancer (145). One study found that in human glioma cell lines and primary tumors, CXCR4 expression correlated directly with malignancy. CXCR4 activation induced tumor cell chemotaxis and increased the production of vascular endothelial growth factor. Additionally, glioma cells expressing higher levels of CXCR4 formed more rapidly growing and lethal tumors in nude mice (146). A poorer prognosis was observed after surgery in patients with CXCR4-positive gliomas (137).

^68^Ga-Pentixafor was developed as a radiolabeled cyclic pentapeptide with high affinity to CXCR4 (147). The researchers found that ^68^Ga-Pentixafor PET imaging would be a promising radiotracer for the management of treatment planning in high-grade gliomas. Lapa et al. demonstrated that non-invasive imaging of CXCR4 in human malignant glioma using ^68^Ga-Pentixafor was feasible (147). They showed that ^68^Ga-Pentixafor PET in high-grade glioma yielded positive and receptor expression of malignant cells could be confirmed by immunohistochemical workup. Due to the extremely low background activity uptake of ^68^Ga-Pentixafor in the brain, it is very favorable and has a significantly higher tumor uptake compared to amino acid tracers such as ^18^F-FET, which confirms the high specificity of the tracer. In this regard, radioligand agents such as ^177^Lu in combination with Pentixafor seem to be a promising new approach for glioblastoma therapy. CXCR4-directed therapy alone or with temozolomide has already yielded promising results.

**Figure 2:**
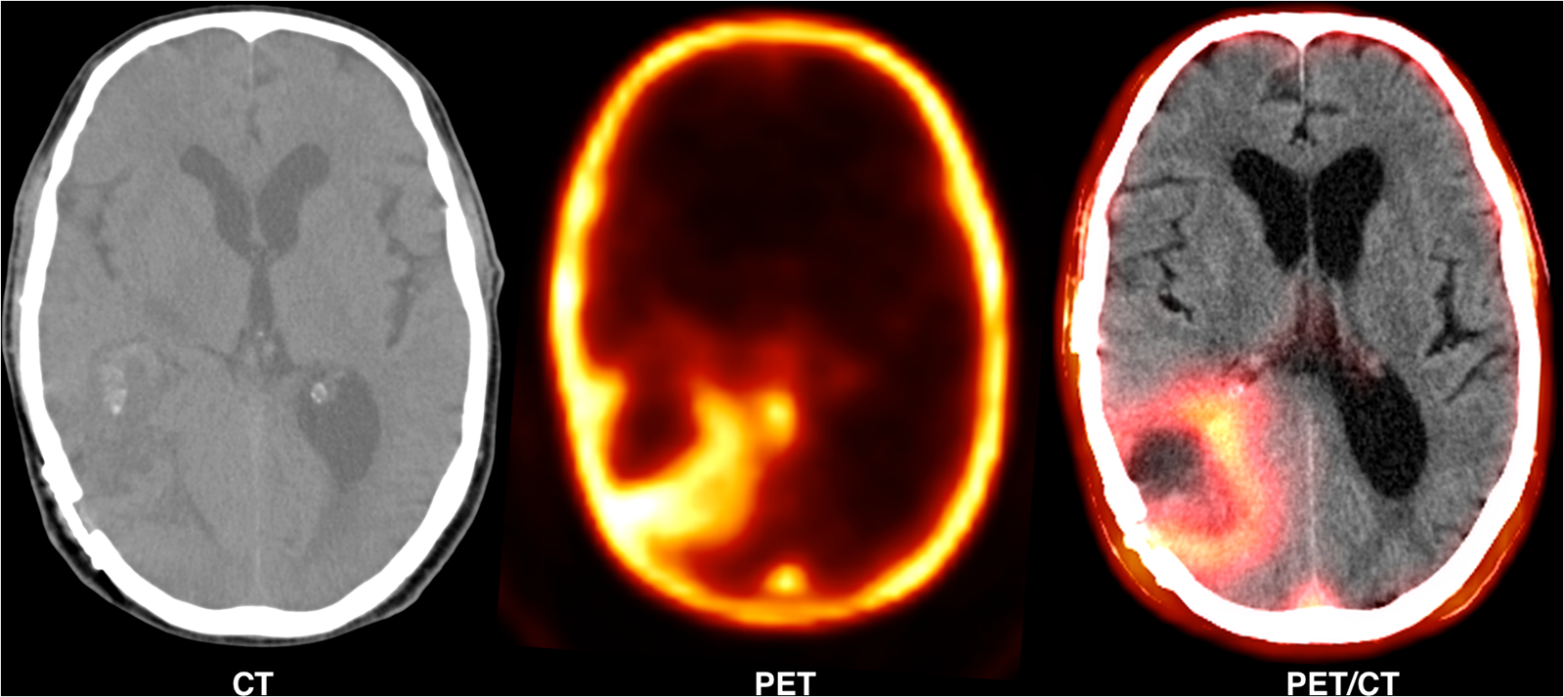
A 62-year-old man with high-grade glioma (WHO IV) that underwent ^68^Ga-pentixafor PET/CT. A large hypo-dense cerebral lesion (7*5.5 cm) was observed in the right parieto-occipital lobe with ^68^Ga-Pentixafor (Pars-Cixafor^TM^) uptake (SUVmax: 1.87), central photopenia (probably necrosis) and good tumor delineation.

**Figure 3:**
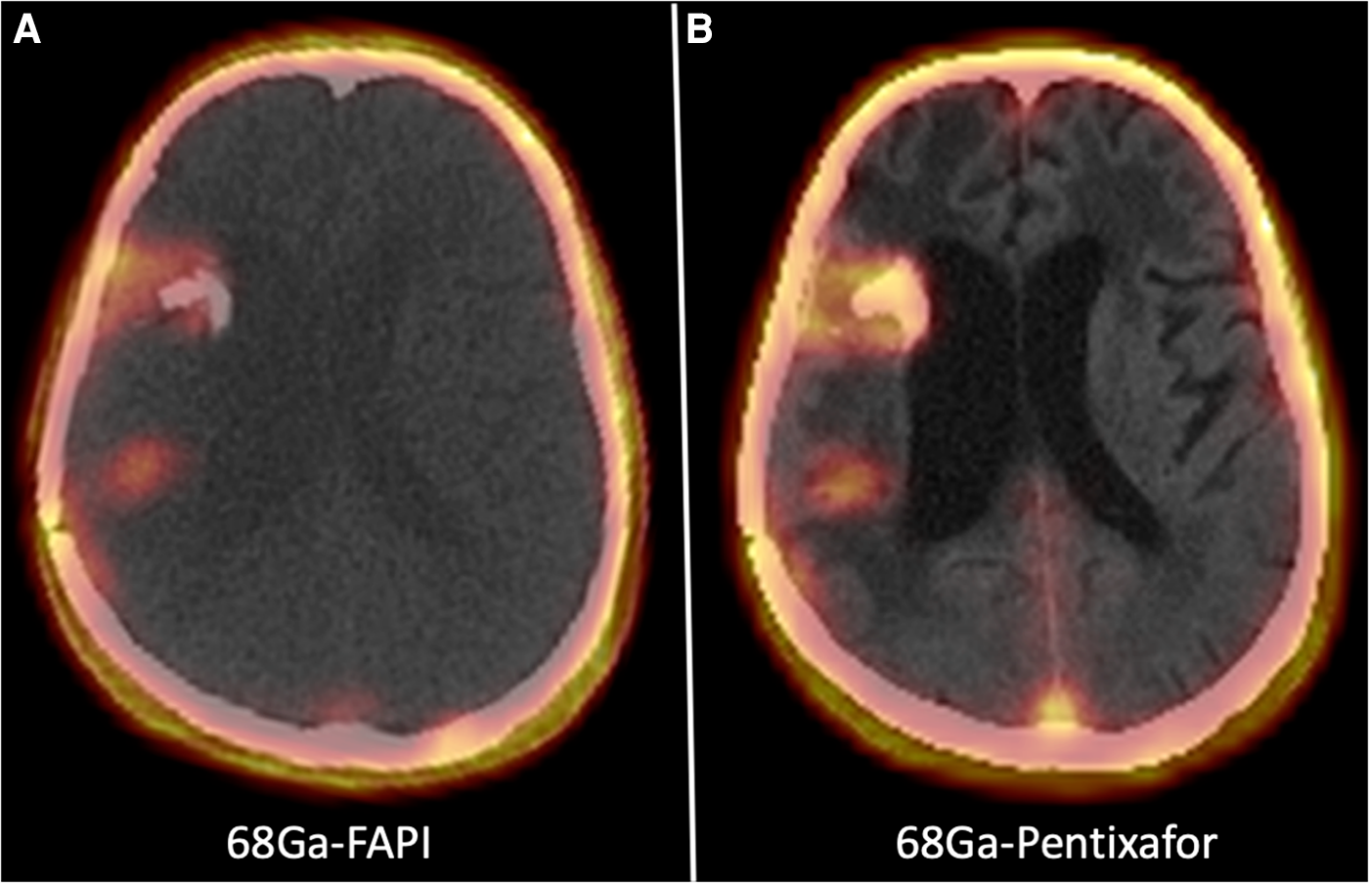
A 55-year-old patient with a history of GBM in the right frontotemporal lobe that underwent surgery and external beam radiation therapy and received ^177^Lu-DOTATATE. On ^68^Ga-Pentixather and ^68^Ga-FAPI imaging, there were three cerebral mass lesions in the right frontal and temporoparietal lobs, including a 11 × 15 mm lesion in the temporal lobe with SUV max = 4.15 (^68^Ga-Pentixather) and SUVmax = 4.154 (^68^Ga-FAPI), a 27 × 15 mm lesion in the temporal lobe (peri ventricular) with SUVmax = 1.97 (^68^Ga-Pentixather) and SUVmax = 2.68 (^68^Ga-FAPI), and a third lesion with a diameter of 12 × 14 mm in the right temporal lobe with SUVmax = 2.23 (^68^Ga-Pentixather) and 1.82 (^68^Ga-FAPI).

Figure 2 shows a 62-year-old man with high-grade glioma who underwent ^68^Ga-CXCR4 PET/CT. A large hypo-dense cerebral lesion with good tumor delineation was observed in the right parieto-occipital lobe. Figure 3 demonstrates ^68^Ga-Pentixather and ^68^Ga-FAPI imaging in a 55-year-old patient with a history of GBM in the right frontotemporal lobe that underwent surgery and external beam radiation therapy and received ^177^Lu-DOTATATE.

### Somatostatin receptor

As a cyclic neuropeptide, somatostatin is well known in nuclear medicine for diagnosis and treatment of neuroendocrine tumors (148). The majority of the human tumors overexpress one or more of the five distinct human SSTR subtype receptors (149). Based on preliminary results from the MAURITIUS trial, of 43 glioblastoma patients treated with 400 to 3,700 MBq of ^90^Y-DOTA-lanreotide in fractionated one-to-six cycles, 5 cases showed regressive disease (reduction of more than 25% of tumor size), 14 cases showed stable disease, and 24 patients had progressive disease (increase of more than 25% of tumor size). In addition, 5 patients reported a subjective improvement in the quality of life measures (150). The authors reported that preclinical data and clinical studies confirmed the promise usefulness of radiolabeled lanreotide for tumor diagnosis and therapy. A case study reported that locally injected ^90^Y-DOTATOC in three patients with grade IV recurrent glioblastoma was feasible and well tolerated. An epileptic seizure recurred in one patient and a transient mild headache occurred on the day of the radiopharmaceutical application in another patient. This approach showed an attractive strategy for treating locally recurring or progressing glioblastoma (151).

### Prostate-specific membrane antigen (PSMA)

PSMA is a type II transmembrane glycoprotein. Its overexpression is positively associated with higher tumor grades and stages in prostate cancer. PSMA expression is not limited to prostate cancers. Some tissues, such as salivary glands, express PSMA physiologically, and monoclonal antibodies to the extracellular domain of PSMA are also highly reactive with tumor vasculature in a variety of cancers, such as lung, breast, and colon carcinoma (152, 153). In one study, a single case of a glioblastoma patient was analyzed in whom the tumor also expressed PSMA on its neo-vasculature (154). Multiple immunohistopathological studies have confirmed PSMA expression in the tumoral vessels of high-grade gliomas (155–159).

With the development of ^68^Ga-labeled PSMA, a PSMA-targeted small molecule labeled with ^68^Ga, the researchers also use it as a therapeutic target with different radionuclides, e.g., ^177^Lu. ^68^Ga-PSMA is increasingly used in the routine clinical assessment of prostate cancer patients in the initial staging of intermediate or high-risk prostate cancers and in restaging after biochemical clue of tumor recurrence. With its extremely low background uptake in the normal brain tissue and a high tumor-to-brain ratio, ^68^Ga-PSMA-11 PET/CT is highly promising for the diagnosis of recurrent GBM (160). Nevertheless, a low tumor-to-liver ratio observed in a study by Kunikowska et al. suggested that radiolabeled PSMA ligands may have a limited role in targeted radionuclide therapy of recurrent GBM (160). In 2021, a re-resection of a recurrent tumor following radiochemotherapy and subsequent chemotherapy was performed in 16 patients with de-novo glioblastoma (161). The results showed that both the initial diagnosis and recurrence of glioblastoma exhibited PSMA expression. The authors argued that high vascular PSMA expression upon recurrence appeared to be associated with a poor prognosis; therefore, PSMA expression in recurrent GBM might serve as a promising target for theranostic approaches. The authors recommended further studies on PSMA PET imaging and PSMA-directed radioligand therapy in patients with brain tumors. PSMA PET imaging was used to assess the potential of PMSA radioligand therapy in syngeneic GL261 GBM models in one study (162). The results showed that although ^18^F-PSMA-1007 PET imaging of GL261 tumor-bearing mice was feasible and produced high tumor-to-background ratio, absolute tumor uptake values remained low, suggesting the limited application of the GL261 model for PSMA-directed therapy studies.

### Fibroblast activated protein

During recent decades, numerous radiotracers and their application in neuro-oncology have been investigated in gliomas. Functional imaging techniques, as complementary tools to structural MRI, are highly needed for tumor entities such as anaplastic astrocytoma and GBM (163). As discussed earlier, while amino acid-based tracers such as FET have shown good results in comparison with FDG, other promising radiotracers such as fibroblast activated protein inhibitors (FAPIs) can also detect tumors based on the expression of fibroblast activation protein in the tumor stroma in cancer-activated ﬁbroblasts of GBM.

Various types of FAPI have been already established such as FAPI-02/04-46/74 and their biodistribution in GBM patients is currently under investigation (164, 165). As a pioneer researcher in the production of FAP, Giesel et al. assessed the biodistribution of ^68^Ga-FAPI-02/04 in eight patients with head and neck cancer (166). The results showed that tumor-to-background ratios of FAP-specific PET were almost similar to ^18^F-FDG-PET. Röhrich et al. showed increased FAPI-02/04 uptake in 13 patients with grade III/IV gliomas and high-grade glioblastomas (167). Additionally, Chen et al. compared ^68^Ga-DOTA-FAPI-04 and ^18^F-FDG PET/CT for the diagnosis of primary and metastatic lesions in patients with various types of cancer including four glioma patients (two GBM, one grade II glioma, and one grade III glioma). However, the absolute uptake of FAP for tumor was low and the tumor-to-background ratio was high, lower uptake of FAP with a higher tumor-to-background ratio were observed in gliomas in other brain studies (168–170).

A case report study by Ballal1 et al. showed the possibility of a theranostic approach of ^68^Ga-DOTA.SA.FAPi PET/CT-guided ^177^Lu-DOTA.SA.FAPi radionuclide therapy in an end-stage breast cancer patient with confirmed brain metastasis (171). The patient received a single cycle of ^177^Lu-DOTA.SA.FAPi. There was physiological uptake of ^177^Lu-DOTA.SA.FAPi in the liver, kidneys, pancreas, and background muscle, and an intense accumulation of radiotracer was noted in all lesions in agreement with ^68^Ga-DOTA.SA.FAPi PET/CT scans. The authors declared that in patients not responding to conventional treatment options, ^68^Ga-DOTA.SA.FAPi PET/CT-guided ^177^Lu-DOTA.SA.FAPi therapy might offer a new opportunity for a theranostic approach in breast cancer therapy.

Moon et al. reported better tumor retention by introducing squaric acid (SA) as a linker into the FAP inhibitor yielding DATA5m.SA.FAPi and DOTA.SA.FAPi (172). Wang et al. demonstrated that ^68^Ga-DOTA.SA.FAPi PET/CT and ^18^F-FDG PET/CT had similarly high SUL uptake values in the primary site of head and neck cancers (173). In another study by Ballal et al. ^68^Ga-DOTA.SA.FAPi identified additional lesions in the brain that could not be detected on ^18^F-FDG PET/CT (174). According to the results, lower cortex uptake of ^68^Ga-DOTA.SA.FAPi in the brain makes it an ideal radiotracer where ^18^F-FDG PET/CT fails to detect brain tumors.

**Figure 4:**
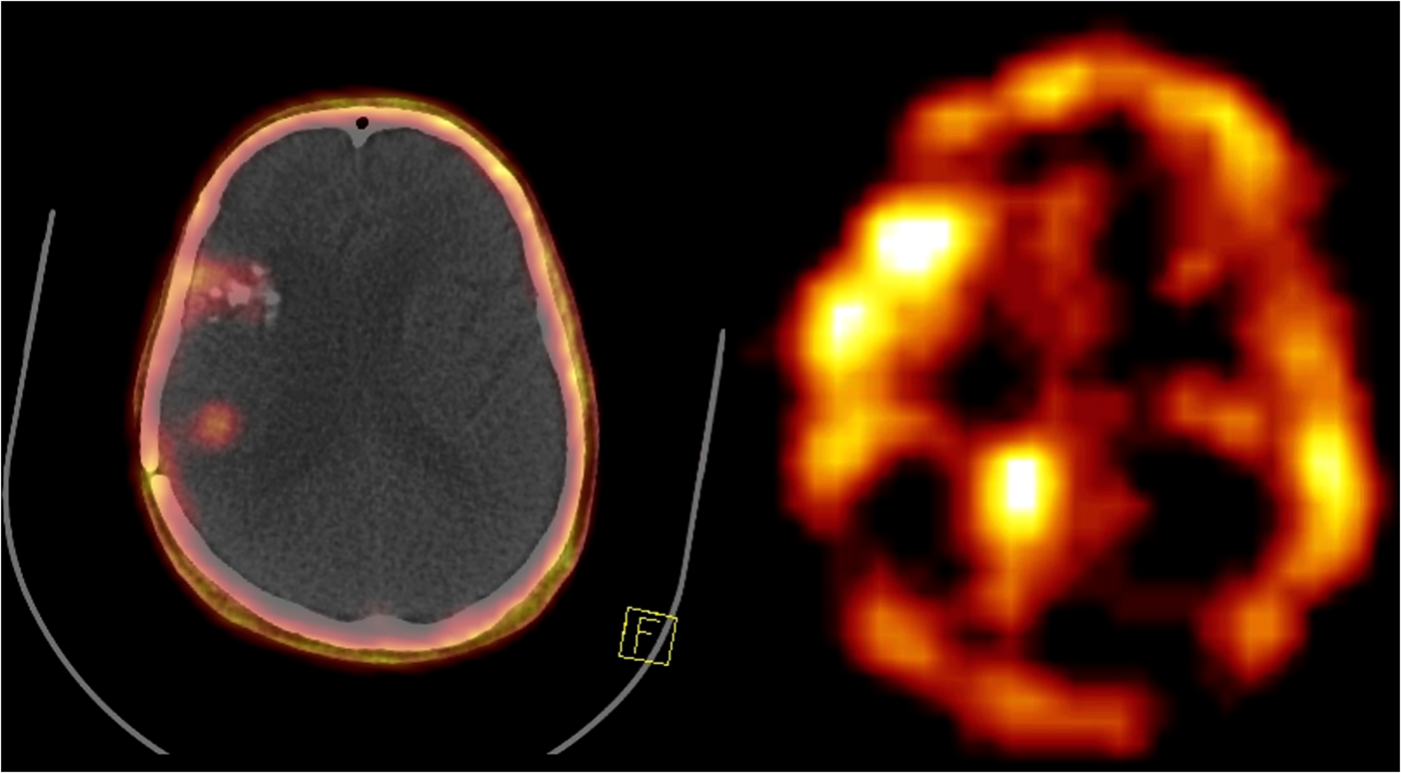
A 55-year-old man with recurrent high-grade glioma in the right temporoparietal region underwent ^68^Ga-FAPI PET/CT showing three cerebral lesions in the right frontal and right temporoparietal regions (left side). The patient received one cycle of ^177^Lu-FAPI (right side).

Figure 4. A 55-year-old man with recurrent high-grade glioma tumor undergoing ^68^Ga-FAPI PET/CT (left side) and one cycle of ^177^Lu-FAPI (right side).

## Conclusion

Glioblastoma multiforme is a fast-growing, invasive brain tumor that is typically associated with fatal outcomes. In recent years, new PET biomarkers have opened new horizons to evaluating a wide range of biochemical processes in terms of the diagnosis, staging, treatment planning, and monitoring disease progression and response to therapy of GBM. Along with the therapeutic potential of nuclear medicine and its successful theranostic applications in different malignancies, it could serve as an additional therapeutic option as neurotheranostics for GBM. Although the majority of these compounds require further clinical investigation, they may have a great potential for combined therapies.
